# Association of Perceived Electronic Health Record Usability With Patient Interactions and Work-Life Integration Among US Physicians

**DOI:** 10.1001/jamanetworkopen.2020.7374

**Published:** 2020-06-22

**Authors:** Edward R. Melnick, Christine A. Sinsky, Liselotte N. Dyrbye, Mickey Trockel, Colin P. West, Laurence Nedelec, Tait Shanafelt

**Affiliations:** 1 Department of Emergency Medicine, Yale University School of Medicine, New Haven, Connecticut; 2 American Medical Association, Chicago, Illinois; 3 Department of Medicine, Mayo Clinic, Rochester, Minnesota; 4 Department of Psychiatry and Behavioral Sciences, Stanford University, Palo Alto, California; 5 Department of Health Sciences Research, Mayo Clinic, Rochester, Minnesota; 6 Department of Medicine, Stanford University School of Medicine, Palo Alto, California

## Abstract

This cross-sectional survey assesses the association of perceived electronic health record usability with patient interaction and work-life integration among US physicians.

## Introduction

Electronic health record (EHR) usability is unacceptable to most US physicians and has been reported to be inversely associated with professional burnout,^1,2,3^ yet to date, little is known about EHR usability in terms of patient interaction and work-life integration. In this artcile, we assess the associations of perceived EHR usability with patient interaction and work-life integration.

## Methods

This cross-sectional survey sampled 870 US practicing physicians from all specialty disciplines represented in the American Medical Association Physician Masterfile between October 2017 and March 2018. Full sampling details and assessment for response bias have been previously reported.^4^ A random 25% of responders also received an EHR subsurvey. The Stanford University and Mayo Clinic institutional review boards approved the study protocol. Participation in the voluntary anonymous survey was considered implied consent. This study followed the Strengthening the Reporting of Observational Studies in Epidemiology (STROBE) reporting guideline for cross-sectional studies.

The role of EHRs in physician-patient interactions and work-life integration was assessed with 4 items, 2 each for perceived benefit and perceived disadvantage (Figure and Table). Individuals who indicated often or very often to the EHR benefit items or never or rarely for the EHR disadvantage items were considered to be satisfied with their EHR’s integration in patient care and home and work life. Perceived EHR usability was measured using the system usability scale (SUS), a short, reliable usability industry standard (score range, 0-100; higher scores indicate greater satisfaction with usability).^5^ Multivariable logistic regression was performed to identify characteristics associated with satisfaction with the EHR with regard to patient care and work-life integration. A 2-tailed *P* < .05 was the level at which statistical significance was set. All analyses were completed using R statistical software, version 3.5.3 (R Project for Statistical Computing).

**Figure.**
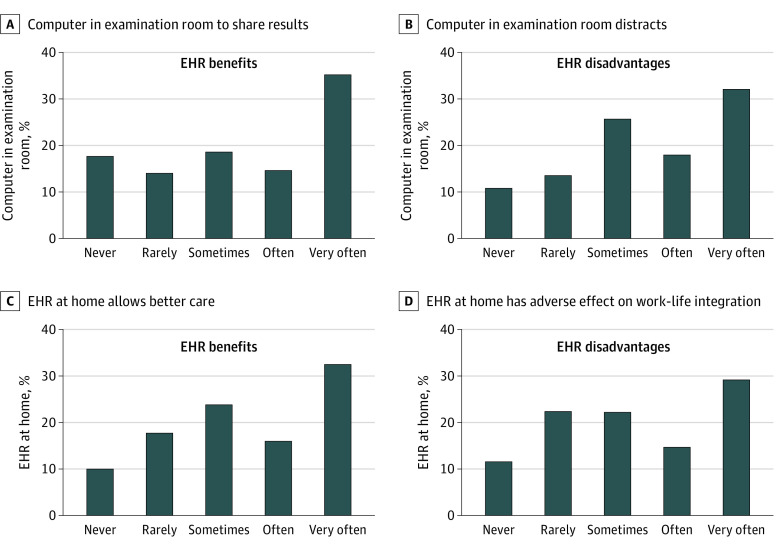
Distribution of Survey Responses on Electronic Health Record (EHR) Integration Into Home and Work Life

**Table.** Factors Associated With EHRs and Patient Care and Work-Life Integration Outcomes Among Practicing Physicians in 2017

Survey item	Response	Factor	OR (95% CI)	*P* value
EHR benefit				
“I use a computer in the exam room (or at the bedside) to share test results with my patients”	Often or very often	SUS	1.01 (1.00-1.02)	<.001
		Veterans hospital setting	4.96 (1.36-18.11)	.02
		Nonprimary care	0.42 (0.30-0.56)	<.001
“Access to the EHR while at home allows me to provide better care to my patients”	Often or very often	SUS	1.03 (1.02-1.04)	<.001
EHR disadvantages				
“Interacting with a computer in the exam room (or at the bedside) distracts me from interacting with my patients”	Never or rarely	SUS	1.04 (1.03-1.05)	<.001
		Nights on call per week	0.87 (0.79-0.96)	.005
		Nonprimary care	1.73 (1.13-2.64)	.01
“Interacting with the EHR while at home has had an adverse effect on my work-life integration”	Rarely or never	SUS	1.02 (1.02-1.03)	<.001
		Nonprimary care	1.77 (1.19-2.62)	.004

Abbreviations: EHR, electronic health record; OR, odds ratio; SUS, system usability scale (score range, 0-100; higher scores indicate greater satisfaction with usability).

## Results

The analysis included 870 respondents (353 female [40.6%]; median [interquartile range] age, 53 [42-61] years). Full demographic characteristics and results by specialty were previously reported.^3^ Approximately half of respondents (49.8%) indicated that having a computer in the examination room allowed them to share test results with patients often, whereas nearly half (43.9%) also indicated that having a computer in the examination room was often distracting (Figure). Although approximately half of respondents (50.1%) felt that EHR access when at home allowed better care, a large proportion (43.9%) also felt EHR access at home often had an adverse effect on work-life integration. On multivariable analysis adjusting for age, sex, hours worked per week, number of nights on call per week, practice setting, primary care, and marital status, every 1 point higher EHR SUS score was associated with a higher likelihood of satisfaction with (1) computer use in the examination room to share test results with patients (odds ratio [OR], 1.01; 95% CI,1.00-1.02; *P* < .001) (Table) and (2) access to the EHR at home to allow better care (OR, 1.03; 95% CI, 1.02-1.04; *P* < .001). In addition, each 1 point higher SUS score was associated with a higher likelihood of reporting that (1) the computer in the examination room rarely or never distracts from interacting with patients (OR 1.04; 95% CI, 1.03-1.05; *P* < .001) and (2) EHR access at home rarely or never has an adverse effect on work-life integration (OR 1.02; 95% CI, 1.02-1.03; *P* < .001).

## Discussion

We found that physicians recognize both the value of the EHR to patient care and negative associations with patient interactions and work-life integration.^6^ Furthermore, higher physician perceived EHR usability was associated with higher levels of perceived positive outcomes (improved patient care) and lower levels of perceived negative outcomes (worse patient interactions and work-life integration). Limitations include that a cross-sectional study is unable to determine causation or the direction of effect. We believe our findings give cause for optimism. Usability can be improved if prioritized by those who design, implement, and regulate EHRs, and prioritizing usability may help improve patient care and physician well-being. We also believe clearer boundaries are needed to protect against the invasive nature of EHRs in creating work outside of the workday. Better usability and clearer boundaries will likely support therapeutic relationships between physicians and patients and the well-being of the physician workforce.

## References

[1] Friedberg MW, Chen PG, Van Busum KR, Factors affecting physician professional satisfaction and their implications for patient care, health systems, and health policy. Rand Health Q. 2014;3(4):1. 28083306 10.7249/rhq-03-04-01 PMC5051918

[2] Shanafelt TD, Dyrbye LN, Sinsky C, Relationship between clerical burden and characteristics of the electronic environment with physician burnout and professional satisfaction. Mayo Clin Proc. 2016;91(7):836-848. doi:10.1016/j.mayocp.2016.05.007 27313121

[3] Melnick ER, Dyrbye LN, Sinsky CA, The association between perceived electronic health record usability and professional burnout among US physicians. Mayo Clin Proc. 2020;95(3):476-487. doi:10.1016/j.mayocp.2019.09.024 31735343

[4] Shanafelt TD, Hasan O, Dyrbye LN, Changes in burnout and satisfaction with work-life balance in physicians and the general US working population between 2011 and 2014 [published correction appears in *Mayo Clin Proc*. 2016 Feb;91(2):276]. Mayo Clin Proc. 2015;90(12):1600-1613. doi:10.1016/j.mayocp.2015.08.023 26653297

[5] Brooke J SUS: a retrospective. J Usability Stud. 2013;8(2):29-40. https://uxpajournal.org/wp-content/uploads/sites/8/pdf/JUS_Brooke_February_2013.pdf

[6] Gellert G, Webster L, Gillean J, Melnick E, Kanzaria H Should US doctors embrace electronic health records? BMJ. 2017;356:j242. 28119282 10.1136/bmj.j242

